# The Hygiene of the Home

**Published:** 1891-04

**Authors:** 


### HALL'S JOURNAL OF HEALTH

TRUTH DEMANDS NO SACRIFICE; ERROR CAN MAKE NONE.

Vol. 38. APRIL, 1891. No. 4.

### THE HYGIENE OF THE HOME.

The first consideration of the home should be in reference to its healthfulness. It is only by a due attention to particulars, that the household is maintained at its best, and this includes not only the indoor arrangements, but the immediate outdoor surroundings. That "order is heaven's first law" has passed into a proverb, and order embraces fitness, arrangement, simplicity and above all neatness. It should begin with the cellar and end nowhere. Too often is it the case that certain unfrequented rooms, especially those below ground and under roof, are quite overlooked on the occasion of periodical house-cleanings, and yet the air of the living rooms is made foul by emanations of decayed vegetables from below, and the accumulated dust of the lumber garret is a standing invitation to the pestiferous microbe.

In populous cities the drain pipes and sewers are a prolific source of disease, to repel which, even the most efficient safeguards are incomplete, but much is gained to health by keeping the run clear of obstruction, with an occasional flushing, with the addition of lime, soda, or salt, to hot water.

The back yard, however ample or contracted, should never be used as the depository of *rubbish*. On moving or clearing out days, we look upon the dusty pile of worn out and utterly useless truck brought to light, and wonder where it all came from. Not one thing of all is worth saving or carrying away, and the whole obtruding collection of good-for-nothings should have been long before discarded from the family storehouse.

We are apt to be too indifferent in respect to the environments of our country homes, which are not accessible to any general system of sewage. All waste water should be carried a considerable distance from the house, and never suffered to stagnate in the open air. For want of better means it is customary to lead it in drain pipes to a covered cess-pool, but this method, oftentimes the most convenient and economical, is a continual menace to healthfulness, for the neighboring earth soon becoming saturated, sends off its inordinate moisture, in poisonous exhalations from the surface. If the water supply is from a well, it should be located beyond the possible impregnation of objectionable deposits. We have in mind the corruption of well water—in one instance by a cow yard and in another by a petroleum refinery, located at a distance of a hundred feet or more. The trouble may be years in manifesting itself, but it is sure to come in time. Open wells in frequent use are preferable to closed ones, inasmuch as they have the advantage of continual fresh air purification.

The needful appurtenances of a country home are a series of out buildings, including a stable, cow shed, hen house, etc. Whilst these should be conveniently accessible from the dwelling, they should never be suffered to encroach upon its sanitary requirements. Good health is the foremost consideration, and nothing should be allowed to stand in the way of it. In this regard a great responsibility rests upon parents, which the exercise of prudence and sound common sense will wisely meet, and gratefully fulfill.

